# The INSPIRE Population Survey: development, dissemination and respondent characteristics

**DOI:** 10.1186/s12874-021-01329-3

**Published:** 2021-06-24

**Authors:** Flaka Siqeca, Katrina Obas, Olivia Yip, Samuel Stenz, Penelope Vounatsou, Matthias Briel, Matthias Schwenkglenks, Carlos Quinto, Eva Blozik, Andreas Zeller, Leah L. Zullig, Sabina De Geest, Mieke Deschodt

**Affiliations:** 1 Department Public Health, Institute of Nursing Science, University of Basel, 4051 Basel, Switzerland; 2 Department of Epidemiology and Public Health, Swiss Tropical and Public Health Institute, 4051 Basel, Switzerland; 3 Department of Clinical Research, Basel Institute for Clinical Epidemiology and Biostatistics, University Hospital Basel, 4051 Basel, Switzerland; 4 Department Public Health, Institute of Pharmaceutical Medicine (ECPM), University of Basel, 4051 Basel, Switzerland; 5 Aerztegesellschaft Baselland, 4132 Muttenz, Switzerland; 6 Helsana-Gruppe, 8001 Zürich, Switzerland; 7 Department Clinical Research, Center for Primary Health Care, University of Basel, 4051 Basel, Switzerland; 8 Department of Population Health Sciences, Duke University School of Medicine, Durham, NC 27701 USA; 9 Department of Public Health and Primary Care, Gerontology and Geriatrics, KU Leuven, 3000 Leuven, Belgium

**Keywords:** Aged, Delivery of healthcare, Integrated, Demographic Survey, Surveys and Questionnaires, Community-care, Stakeholder involvement

## Abstract

**Background:**

Most older adults prefer to continue living at home despite increasing care needs and demand for services. To aid in maintaining independence, integrated care models for community-dwelling older people are promoted as the most cost-effective approach. The implementation of such care models is challenging and often the end-users are not involved or their needs are not considered. We conducted a population survey in order to understand the needs and preferences of home-dwelling older adults living in Canton Basel-Landschaft, Switzerland. The aims of this paper are to chronicle the development of the INSPIRE Population Survey, outline its variables and measurements, describe the marketing strategy utilized for survey dissemination and report on the response rate and respondent characteristics.

**Methods:**

The INSPIRE Population Survey, conducted between March and August 2019, is a cross-sectional survey of older adults aged 75 and older living at home in Canton Basel-Landschaft. The questionnaire was developed by expert input and stakeholder involvement. Its readability and acceptability were pilot-tested with older people. To ensure the likelihood of a high and representative response rate, a meticulous step-by-step marketing strategy was developed prior to the dissemination of the questionnaire.

**Results:**

The overall response rate was 30.7% (*n* = 8,846), with variations between 20.6 and 34.5% across the different care regions in the canton. A generally higher response rate was found in the care regions with a higher density and which bordered the urban city of Basel. We received support from local stakeholders, policy makers and media through using a broad combination of marketing channels and targeting our community partners who have a strong relationship with our target audience.

**Conclusions:**

Although recruiting older adults in research is challenging, our study shows that a high response rate can be achieved by developing the survey through expert input and by involving all important stakeholders, including older adults, throughout the entire process.

**Supplementary Information:**

The online version contains supplementary material available at 10.1186/s12874-021-01329-3.

## Background

With older age, the health and social needs of older adults become more complex and the demand for services and associated costs increases [1]. Older adults are considered to be at a higher risk of developing geriatric syndromes, such as delirium, falls, incontinence and frailty [2]. Furthermore, multimorbidity, the coexistence of two or more chronic diseases, is prevalent between 62 to 81% among adults older than 65 years [3]. Despite the potential age-related decline in basic and instrumental activities of daily living, most older adults prefer to maintain their independence and continue living at home [4]. Because older people often have multiple health needs, services designated to treat a single disease or demand are not optimal [5–7]. In contrast, integrated approaches to service delivery can be more cost-effective and result in better outcomes for community-dwelling older adults [8]. The World Health Organization has defined integrated care services as services managed and delivered in order for people to receive a continuum of care, coordinated across the different levels within and beyond the health sector and tailored to their individual needs [9].

Implementing integrated care services is rather challenging because they frequently include many intervention components, target several outcomes and occur at various system levels [10, 11]. While they often involve different types of health professionals, policy makers and other stakeholders, the end-users are often overseen, despite the strong promotion of involving them in the design, delivery and implementation of integrated service research [12]. There are several acknowledged benefits of involving older adults as end-users to better understand their needs and preferences [12]. These include facilitating of implementation of health technologies, clarification of areas of practice that can improve care, enhancing the quality of research as well as improving chances of conducting more relevant research [12]. Hence, in order to transform the system to support integrated care, engaging older adults in healthcare research and planning is essential [13].

Consistent with the recommendations to involve older people as important stakeholders in building better coordinated systems [9], the INSPIRE project (ImplemeNtation of a community-baSed care Program for home dwelling senIoR citizEns) is striving to develop and implement an integrated care model for people aged 75 and older living at home in Canton Basel-Landschaft (BL) in Switzerland. It is a project positioned within the first three phases of the Medical Research Council (MRC) framework for developing and evaluating complex interventions, all the while integrating implementation science components [see Additional file 1] [14]. During the first phase – the development phase—an understanding of the context aids in ensuring the suitability of the intervention components for the implementation setting [15, 16]. Consistently, the INSPIRE Population Survey was conducted as part of the contextual analysis of the INSPIRE project, with the principal aim to gain a better understanding of both current as well as anticipated needs and preferences of people aged 75 and above living at home in Canton BL.

The objectives of the current paper are to (1) chronicle the development of the INSPIRE Population Survey; (2) outline the variables and measurements used in the survey; (3) describe the marketing strategy utilized for the dissemination of the survey and (4) report on the response rate and respondent characteristics. The paper is specifically focused on the development, dissemination and respondents’ characteristics and it is not intended to serve as a paper reporting on the overall results of the survey. Therefore, it is structured in a manner that the methods section is placed central to describe the way the survey questions were developed and which dissemination approach was utilized.

## Methods

### Design, sample and setting

The INSPIRE Population Survey is a cross-sectional study conducted between March and August 2019 in Canton BL in Switzerland, a German-speaking region with a mixture of urban and rural areas. All home-dwelling older adults who were 75 or older and living at home in this region were invited to participate in the study. Only older adults living in a nursing or a care home were excluded.

**Fig. 1:**
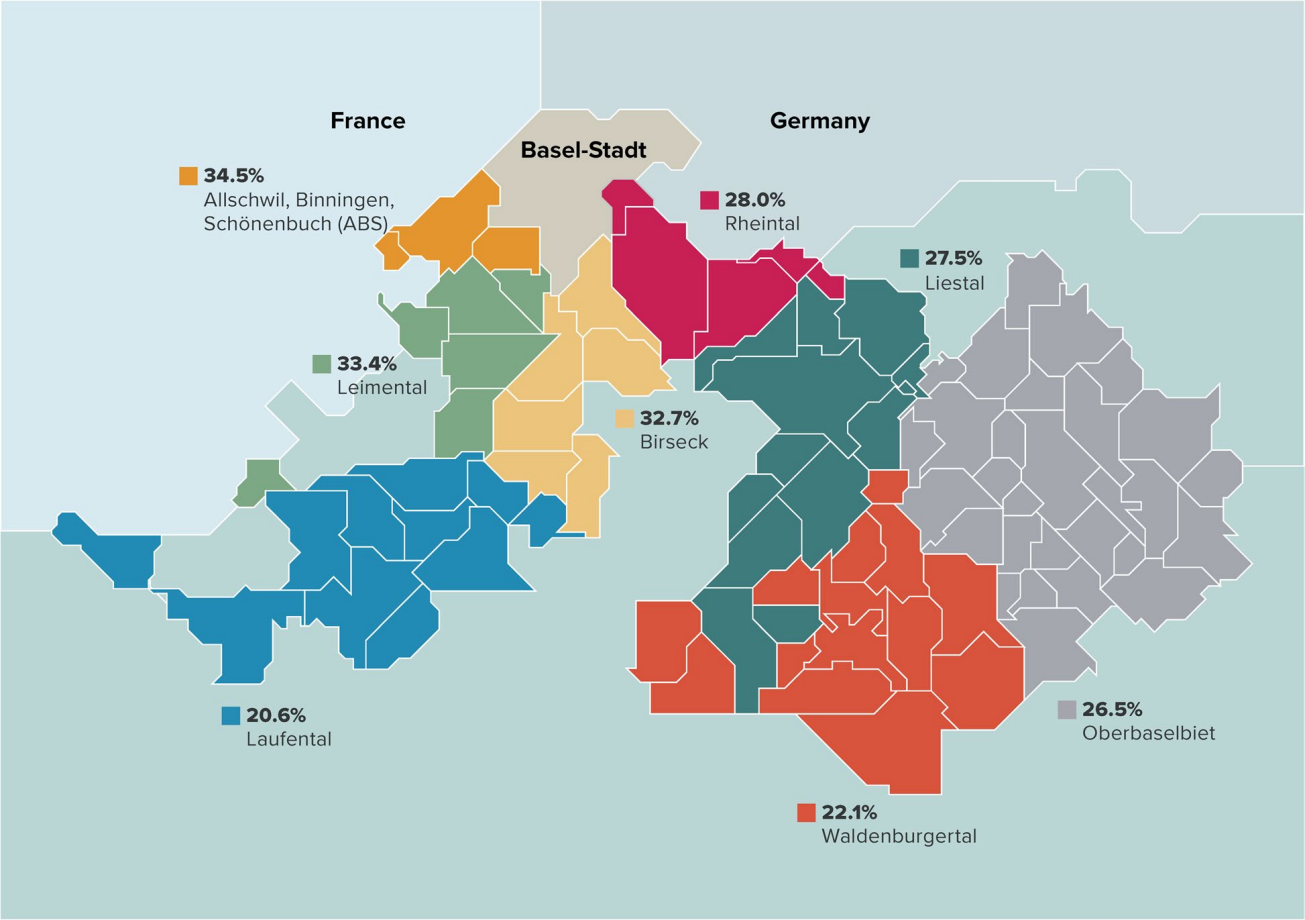
Map of the care regions of Canton Basel-Landschaft and their corresponding response rates

Canton BL is inhabited by 290,000 citizens distributed in 86 municipalities, and has the second highest proportion (21%) of population aged 65 and older in Switzerland [17]. The municipalities were grouped into eight care regions as mandated by a new law published in January 2018 (Fig. 1) [18]. The range of municipalities comprising a given care region varied broadly between only 3 in the Allschwil, Binningen and Schönenbuch (ABS) region to as high as 29 municipalities in the Oberbaselbiet region.

### The development of the INSPIRE Population Survey

The INSPIRE Population Survey was developed through a comprehensive step-wise and iterative process involving various stakeholders. In November 2018, a draft version of the survey containing 154 questions was developed by the research team through a literature review and several internal discussion rounds. Several validated instruments were included in this draft, such as the Groningen Frailty Index [19], the Lawton-Brody scale [20], the Barthel Index of Activities of Daily Living [21], the Brief Social Support Scale [22] and the Satisfaction with Life Scale [23]. This draft version was discussed with an expert group consisting of 11 stakeholders from BL, a heterogenous group of experts with different professional backgrounds and expertise. This group included representatives from the Cantonal Health Department and the association of the BL municipalities; organizations providing health and social services; the association of Swiss nursing homes; the Cantonal hospital; the association of general practitioners of Canton BL and the Ombudsman`s office of Canton BL. These experts considered the questionnaire too long and stated that some questions were intrusive, such as those on independent grooming, bathing, toilet use and dressing coming from the Barthel Index of Activities of Daily Living [21]. Based on this feedback, 71 questions were removed. As the entire INSPIRE project heavily relies on stakeholder involvement, we had to find a balance between the researchers’ needs and the stakeholders’ preferences. The shortened version was e-mailed back to the experts to allow for supplementary input. Additionally, it was also sent for review to the Cantonal Stakeholder Committee which, in addition to the 11 aforementioned experts, also included representatives from various municipalities of Canton BL, representatives of working groups in charge of forming the care regions, as well as representatives from other health and social care providers in the region. Their main feedback was that the survey should not include clinical questions about the presence of chronical illnesses or a list of medications, but should instead mainly focus on the living situation and the needs of this target population. This prompted the removal of an additional 8 questions. This version, approved by all involved stakeholders, was ultimately also pilot-tested for clarity and readability with 6 older adults. One of the largest service provider organizations for senior citizens in Switzerland, which was part of our stakeholder group, supported us with the pilot-testing of the questionnaire. They helped us select a sample of 6 individuals aged 75 and above that were clients of this organization. Minor changes to the clarity of the questions as well as adaptations to answer choices were made following the pilot-testing, to produce the final version of the questionnaire which contained 75 questions.

### The INSPIRE Population Survey

The majority of questions in the final survey were multiple-choice close-ended questions. Questions were formulated to be direct and specific, using the active voice and providing examples to illustrate key information. Special consideration was given to the easily readable font typeface with sufficiently large letters and limited use of italics, underlining or bolding for emphasis, as recommended by the National Institute of Aging of the U.S. Department of Health and Human Services [24]. The questionnaire took approximately 30 to 60 min to be filled out. A detailed description of variables and instruments utilized can be found below whereas an English version of the INSPIRE Population Survey, which has been provided for informative purposes and for which no backward translation was performed, can be found in Additional file 2.

#### Baseline demographics

The demographic information collected included information on age (year of birth); gender; birth country; education level; German language competency level; household income and size; as well as the type of social and health insurance. Information on whether the participant filled-out the questionnaire themselves or with the help of others as well as the degree of urbanization, defined by the postcode, was also collected.

#### Frailty

The Groningen Frailty Indicator (GFI) tool consisting of fifteen questions was embedded within the questionnaire to assess the prevalence of frailty among participants. This instrument is aimed at determining the level of frailty through measuring loss of function in four domains: physical (mobility functions, multiple health problems, physical fatigue, vision and hearing), cognitive (cognitive dysfunction), social (emotional isolation) and psychological (depressed mood and feelings of anxiety) [19]. Answer choices are dichotomized for each question, with a score of 1 indicating a problem or dependency. The GFI score therefore ranges between 0 and 15, with geriatric experts agreeing that a score of 4 or higher represents frailty [19]. The tool has been validated and adapted in German [25].

#### Current and anticipated living situation and arrangements

These questions were developed by the research team to understand what comprises a good living situation for the participants (e.g., access to public transport, proximity to cultural or leisure activities, living in their own house, having a garden, etc.); what is their current living arrangement and household composition; the physical environment of their current living space (e.g., whether they had stairs with handles or whether their bedroom and bathroom were on the same floor) and their overall satisfaction with their current living situation. Furthermore, the anticipated living situation and arrangements were assessed by asking what the ideal living situation would be for them in case of dependency in the future. Most of these questions were categorical and allowed for multiple responses in a single question. Included were also three out of five questions from the Satisfaction with Life Scale, which was validated in German [23]. The answer choices for the three selected questions included the following: “Agree”; “Neither agree nor disagree” and “Disagree”.

#### Health and social services utilization

Healthcare utilization, was assessed by asking participants about the frequency of visits to the general practitioner, specialists, emergency department and hospital overnight stays in the previous year. Additionally, the type of social services they had utilized in the previous year as well as information on the ones they anticipate to need in the future (e.g., meal services, assistance with chores, transport services, etc.) were also captured.

#### Use of technology

Four questions for each of the following types of technology assessed whether participants used telemedicine to communicate with their healthcare provider; used the phone or SMS services to get information and reminders about medication intake; utilized portable medical devices like heart rate and blood sugar monitors or used a help robot for chores and other types of support in their household. An additional question in this category also explored to which type of technology (i.e., telemedicine, phone or SMS, portable devices or help robot) the participants would be open to use in the future.

#### Health-related quality of life

The EQ-5D-5L instrument was used in this survey to assess health-related quality of life [26]. This instrument comprises of a short descriptive questionnaire and a visual analogue scale (VAS) that are cognitively undemanding and take a short time to complete. The descriptive questionnaire comprises of the following five different dimensions of health: mobility, self-care, usual activities, pain/discomfort and anxiety/depression. Each dimension has five response levels of severity, ranging from 1- no problems, 2-slight problems, 3- moderate problems, 4- severe problems to 5- unable to/extreme problems. The respondents were asked to rate their health state by checking the box next to the most appropriate response level of each of the dimensions. A sum score ranging between 5 – 25 was then calculated, where a higher score denotes more problems. The VAS records self-rated health status on a scale from 0 – 100 where the endpoints are labelled ‘The worst health you can imagine (0)’ to ‘The best health you can imagine (100)’.

#### Health status

The health status of the participants was assessed by asking them whether they experienced vision, hearing or memory problems in their daily life, with dichotomized yes/no answer choices. Questions on the quality of sleep in the past four weeks, unintentional weight loss in the past six months as well as frequency and severity of pain were also included in the questionnaire. Furthermore, polypharmacy (defined as taking 4 or more medications at once) as well as the intake and the frequency of pain and sleep medication were recorded. One additional question also assessed whether the participants were able to independently take their medication correctly.

#### Social support

A question with six sub-questions, three of which assess tangible support and three assess emotional support from the Brief Social Support Scale instrument validated in German were used [22]. In this instrument, responses are scored on a 4-point Likert scale ranging from 1- “never” to 4- “always”. Several additional questions also assessed from whom (both individuals and organizations) the participants currently received support from, as well as from whom they anticipate to receive help from, in case of dependency in the future.

#### Functional status

Functional status was assessed using (in)dependence on instrumental activities of daily living (IADL) instrument[27]. The IADL was measured using the Lawton and Brody scale, which measures (in)dependency for eight activities (telephone use, shopping, food preparation, housekeeping, laundry, mode of transportation, medication use and finances)[27]. The scale has been deemed ideal for community-dwelling older adults and its validity and reliability have also been reported [20].

#### Lifestyle

The lifestyle section included questions on frequency and types of physical activities within a typical week; the amount and frequency of alcohol intake within a typical week as well as the current smoking status. Moreover, we included a list of hobbies and activities (e.g., sports, political parties, church gatherings, etc.) which participants could check indicating whether they were active in or wished to be active in.

### The marketing strategy and dissemination approach

To ensure a high and representative response rate to the INSPIRE Population Survey, we developed a wide-ranging marketing strategy before the dissemination of the survey. A designated team including members of the INSPIRE project as well as two administrative staff from the Institute of Nursing Science was in charge of all the marketing and ensured adherence to the strategy.

We issued a joint press release between the University of Basel and the Office of Public Health of Canton BL to help reach a broader audience and advertise the upcoming survey. Additionally, a designated Swiss member of the team continuously communicated with local newspapers, to ensure the information was reaching all municipalities of Canton BL. Continuous information and updates were also posted on the webpage and the social media platforms of the INSPIRE project. Support for marketing was sought by asking stakeholders and collaborators to put up advertising posters and distributing flyers within their premises such as in doctors’ offices; pharmacies; libraries; churches; grocery stores and local supermarkets; banks as well as in community and recreational centers. All social and healthcare organizations who were active in the region were also contacted and asked to put up posters, distribute flyers and forward an e-mail to their clients detailing the goal and the relevance of our study. Moreover, a local organization which provides support and help to the visually-impaired individuals offered to help any visually-challenged participant to fill out the survey. Finally, a phone help line was made available throughout the entire data collection period, in case the participants or their caregivers had any concerns or questions.

### Data collection

Data collection started in March 2019 and was concluded at the end of August 2019. We had initially set the data collection time until the end of May 2019 (3 months) and did receive back more than 95% of the questionnaires during this period. However, in the subsequent weeks we continued to receive around 20–30 more questionnaires per week, so the research team decided to extend the data collection until the end of August to allow for more participants to express their wishes and preferences through the survey. A survey package containing the questionnaire along with instructions for filling it out, an information sheet, a personalized cover letter, a pre-paid return envelope and the informed consent form was mailed to the home address of all community-dwelling persons aged 75 years or older in Canton BL, from the Cantonal Statistical Office. The study information sheet included important information about the target population, the study procedures and the expected results. Participants were informed about the time needed to complete the questionnaire, the voluntary nature of their participation, the possibility to fill out the questionnaire with the help of a proxy and the data protection procedure. Individuals who presumably would be less likely to respond (i.e., non-native German speakers; the very old and frail; those with cognitive impairment, etc.) have been encouraged to respond with the help of a family member, relative or informal caregiver. No financial incentive was provided for participating in the survey. While we sent a reminder to some of our community partners to enquire if they needed additional marketing items, there was no reminder sent directly to the potential survey respondents.

### Data management

All the questionnaires were pseudonymized prior to being delivered with the intent to allow potential follow-up in the future. However, due to concerns of the general public on data security and based on several stakeholder recommendations, we anonymized the questionnaires after having sent them and destroyed all documents containing identifiable information. Data collected in this project are archived following the current Swiss legal requirements for data protection and according to the Ordinance HRO Art. 5. All anonymized survey questionnaires are stored in a secure, password-protected server at the Institute of Nursing Science, University of Basel. Physical copies of the questionnaires will be stored in the locked archives of the Institute of Nursing Science for 10 years and destroyed afterwards.

### Statistical analysis

General descriptive statistics have been computed for the demographic variables. Measures of central tendency (mean, median) and spread (range, standard deviation) were performed as appropriate for continuous data whereas categorical data have been expressed as percentages.

All statistical analyses were conducted using the latest version of SPSS (version 26.0) [28].

## Results

### Response rate

The INSPIRE Population Survey was successfully delivered to 28,791 eligible individuals. We received a total of 8,846 questionnaires back, thus giving us a total response rate of 30.7%. During the validation process, 60 questionnaires were excluded because ZIP codes were from other Cantons, respondents were younger than 75 years old or were residing in a nursing or a care home.

The response rate varied between care regions from as low as 20.6% in the Laufental region to as high as 34.5% in the ABS region (Fig. 1). We noticed a generally higher response rate across the care regions that had a higher density and in care regions bordering the urban city of Basel as compared to care regions located further and considered more rural (Fig. 1).

### The marketing campaign

We received media support in forms of both written and electronic newspapers as well as from several local radio programs. Seventeen of the local newspapers which are frequently read by older adults wrote articles about the survey to encourage participation. Additionally, twenty-four municipalities published information about the INSPIRE Population Survey on their official websites.

We also received support from several health and social service providers in Canton BL. The Canton BL branch of the association of Swiss Nursing Homes wrote a Twitter post about the survey and the expected benefits of understanding the needs and preferences of older adults living in the region. Moreover, a large umbrella non-profit organization which provides outpatient health services as well as support on household and other chores, also promoted the INSPIRE Population Survey on their website. Some of the local branches of this organization also sent out a promoting flyer directly to their clients.

### Demographic characteristics of the participants

The mean age of all respondents was 81.8 (SD = 4.8) years and a little over half of them (51.8%) were female. More than a half of the respondents (50.7%) had completed an apprenticeship (*Ausbildung*) whereas around 10% had a university degree. Around 9% of the respondents reported the total earnings of their household to be less than 3000 CHF per month which is comparable to reports on household monthly income on a national level [29]. On the other hand, around 11% of the respondents reported monthly household earnings to be above 9000 CHF per month. A detailed description of the demographic characteristics as well as information on the number of inhabitants and population density compared across the eight care regions of Canton BL can be found in Table 1.

**Table 1:** Participant characteristics of the INSPIRE Population Survey (total sample of Canton BL with comparisons across the care regions)

Variable	*Canton BL*	*Care region*							
		***ABS***	***Liestal***	***Leimental***	***Laufental***	***Birseck***	***Rheintal***	***Waldenburgertal***	***Oberbaselbiet***
**Bordering Canton Basel-Stadt**	n/a	Yes	No	Yes	No	Yes	Yes	No	No
**Response rate, n (%)**	8786 (30.7)	1503 (34.5)	1031 (27.5)	1336 (33.4)	280 (20.6)	2038 (32.7)	1368 (28.0)	269 (22.1)	752 (26.5)
**Nr. of inhabitants 75 + **	28,622	4371	3770	3995	1361	6216	4884	1180	2845
**Density (sq km)**	560	6900	6080	6283	2399	6340	8220	2587	7631
**Age, mean (SD)**	81.8 (4.8)	82.0 (4.8)	81.8 (4.8)	81.6 (4.6)	81.7 (4.6)	81.8 (4.7)	81.8 (4.9)	81.4 (4.9)	81.5 (4.8)
**Female gender (%)**	51.8	52.3	49.5	51.1	49.8	53.1	53.4	52.4	49.8
**Education (%)**									
No degree	1.0	1.1	1.0	0.5	1.1	0.9	1.3	1.9	0.8
Elementary school	14.8	12.1	14.2	9.1	24.9	13.1	19.7	22.1	19.8
Apprenticeship	50.7	49.6	51.3	48.8	53.1	51.3	51.3	51.1	51.7
Gymnasium	4.4	6.0	3.5	4.7	2.9	4.8	4.2	4.2	2.0
University of Applied Sciences	14.4	13.5	15.5	16.7	9.5	15.0	13.0	9.5	14.6
University	10.3	13.1	10.1	15.3	4.8	10.3	5.7	7.6	7.2
Other	4.5	4.5	4.4	4.8	3.7	4.8	4.8	3.4	3.9
**Income (%)**									
< 3000	9.3	8.4	9.4	4.6	17.4	8.9	9.2	18.4	12.8
3001–6000	36.6	34.4	35.5	29.8	46.4	35.5	42.4	39.6	42.5
6001–9000	26.5	27.2	29.9	27.9	12.1	26.1	28.2	21.6	24.3
> 9000	11.1	11.6	10.7	18.3	8.3	12.6	6.3	5.6	7.4
Do not know	1.1	0.8	1.1	1.3	0.8	1.3	1.1	0.4	1.1
Do not wish to answer	15.4	17.7	13.4	18.2	15.1	15.6	12.9	14.4	11.9

## Discussion

The aim of the INSPIRE Population Survey was to gain a better understanding of needs and preferences of older adults living at home in Canton BL. The purpose of this paper was to describe the development of the questionnaire, the marketing strategy employed prior to its delivery and compare response rates and demographic characteristics of the participants across the newly formed care regions of Canton BL. We achieved an overall response rate of 30.7% on the cantonal level. In a general population study comparing response rates across postal, internet and telephone modes, our response rate is comparable to the one achieved with the telephone mode (30.2%) and considerably higher than the response rate to postal surveys (10.5%) [30]. Hence, through using postal mail as a delivery method without any direct incentive for participation, we achieved a response rate which is much higher than the one reported by Sinclair et al. for the postal surveys [30].

We believe this response rate is particularly excellent given that our target population has been known to be challenging to reach and might have needed additional support to fill out the questionnaire [31]. The overall success of this survey can be credited to a myriad of factors. First, we consider that testing the acceptability and the readability of the survey, tailoring it to the target population as well as continued feedback from both experts and collaborators has made the survey more appropriate for the respondents. Second, we employed a marketing strategy throughout the entire process to ensure the information was being disseminated thoroughly. We believe the real value of our approach was in using the channels most applicable and trusted by our target population, such as local newspapers and service providers. The marketing efforts which started from the beginning of the study and were constantly reassessed, with an emphasis on diverse methods and approaches, have further aided the success of the survey. Additionally, we also involved most stakeholders early on in the process. The time invested into building a relationship and trust with the stakeholders has undoubtedly contributed to the successful conduct of the study. Finally, we believe that we achieved a high response rate because the topic of the survey appeared to be very important to the respondents. We have received many hand-written notes from the participants expressing their gratitude for the opportunity to express their needs and preferences as they continue aging in their homes.

Nevertheless, we do acknowledge that our study comes with sampling limitations, as we targeted the entire eligible population rather than sampling with a probabilistic approach. We used a non-probabilistic sampling method because we aimed at exploring the needs and preferences of a very specific population to aid the implementation of the INSPIRE project. Furthermore, this approach was a request by the stakeholders of the project, as they wished that the analysis be conducted on regional level to allow for mapping of the needs and preferences of this segment of the population to the specific regions. Moreover, because of the anonymous data collection, we had no means of knowing if non-respondents differed from respondents. There exists the possibility that older adults who responded to our survey were healthier and more engaged in social life than the targeted population on average, while the very old, most frail or cognitively challenged may have been less likely to respond, thus subjecting our study to additional selection bias. However, the percentage of frailty among community-dwelling older adults as measured by the GFI in a comparable study population is in line with our observed results (not presented in this manuscript) [32]. Further limitations may stem from the fact that the entire questionnaire was not tested for reliability and validity, but only included a number of validated instruments [19, 22, 26, 27]. Although we tested the face and content validity, other forms of validity testing, and testing of reliability, were outside the scope of this research. The length of the questionnaire might have also discouraged some participants from responding, especially considering that answering survey questions can be both cognitively and physically demanding for older participants [31]. This is particularly the case with older individuals whose poor vision along with potential decline in cognitive abilities can affect their capability to engage in survey research, especially in paper-based questionnaires [33]. One further limitation that might have hindered us in reaching an even higher response rate is that we did not involve older adults in the very early stage of selecting questions. This could have potentially improved the relevance of the questionnaire for them and increased their willingness to respond [34]. Furthermore, even when older adults were involved during the pilot-testing phase, the sample of older adults available for pilot-testing was a small sample of convenience and may not have fully represented the true diversity of this fragment of the Basel-Landschaft population. Another aspect of our study that might be considered a limitation could be the fact that we have not properly evaluated our marketing strategy, but instead made assumptions on its effectiveness based on the response rate achieved. Moreover, the limited opportunity to involve older adults in the development of the marketing strategy might have hindered our study from achieving an even higher response rate.

Unfortunately, some survey questions had to be substantially reduced as we aimed to reach a balance between research and stakeholder perspective. Hence, some of the information that could have provided further valuable insights, such as the presence of comorbidities and information on medication intake, were not captured in our study. However, the broad range of information collected on the social determinants, the assessment of potential predictors of frailty, the self-reported health indicators and health and social resources of these older adults have the potential to enable projections on future needs of this subpopulation. In the upcoming years, this large and unique data set will be used to explore several research questions that will further inform service planning research. This will include the assessment of predictors of health-related quality of life among community-dwelling older adults as well as the assessment of whether an association exists between quality of life and the family structure of the older adult. Several of the variables collected from this survey will also be used to complement and compare outcomes with both the feasibility and implementation studies of the INSPIRE care model [16], such as for example potentially inappropriate medication use in older age or the social support they receive daily.

## Conclusions

We achieved a desirable response rate compared to other population surveys [30], through an early involvement of most stakeholders in both the process of developing the questionnaire as well as during the marketing process. The data collected in this survey will inform the further development of the INSPIRE care model and can serve as a comparison in later evaluation. Additionally, we believe it will also serve the politicians and the local organizations in the community to tailor future health and social services they plan to provide for older adults living and aging at home in Canton BL.

## Supplementary Information

**Additional file 1**. INSPIRE project overview mapped according to the Medical Research Council (MRC) Framework. The figure illustrates how the INSPIRE project is positioned within the first three phases of the Medical Research Council (MRC) framework for developing and evaluating complex interventions

**Additional file 2**. The INSPIRE Population Survey in English. The file includes the INSPIRE Population Survey in English which had been provided for informative purposes and for which no backward translation was done

## Data Availability

The dataset generated and/or analyzed during the current study is not publicly available because the current manuscript is intended to serve as a protocol and an overview of the development, variables and the response rate of the questionnaire. The dataset will be shared with the main results paper planned in the future but is also available from the corresponding author on reasonable request.

## Abbreviations

INSPIRE: ImplemeNtation of a community-baSed care Program for home dwelling senIoR citizEns
Canton BL: Canton Basel-Landschaft
MRC: Medical Research Council
ABS region: Allschwil, Binningen and Schönenbuch region
