# Supplementary material for: The INSPIRE Population Survey: development, dissemination and respondent characteristics
Source: BMC Med Res Methodol. 2021 Jun 24;21:131. doi: 10.1186/s12874-021-01329-3 (PMC8223353; doi:10.1186/s12874-021-01329-3)
Supplement: Supplementary file 1 — Additional file 1. INSPIRE project overview mapped according to the Medical Research Council (MRC) Framework. The figure illustrates how the INSPIRE project is positioned within the first three phases of the Medical Research Council (MRC) framework for developing and evaluating complex interventions [file 12874_2021_1329_MOESM1_ESM.pdf]

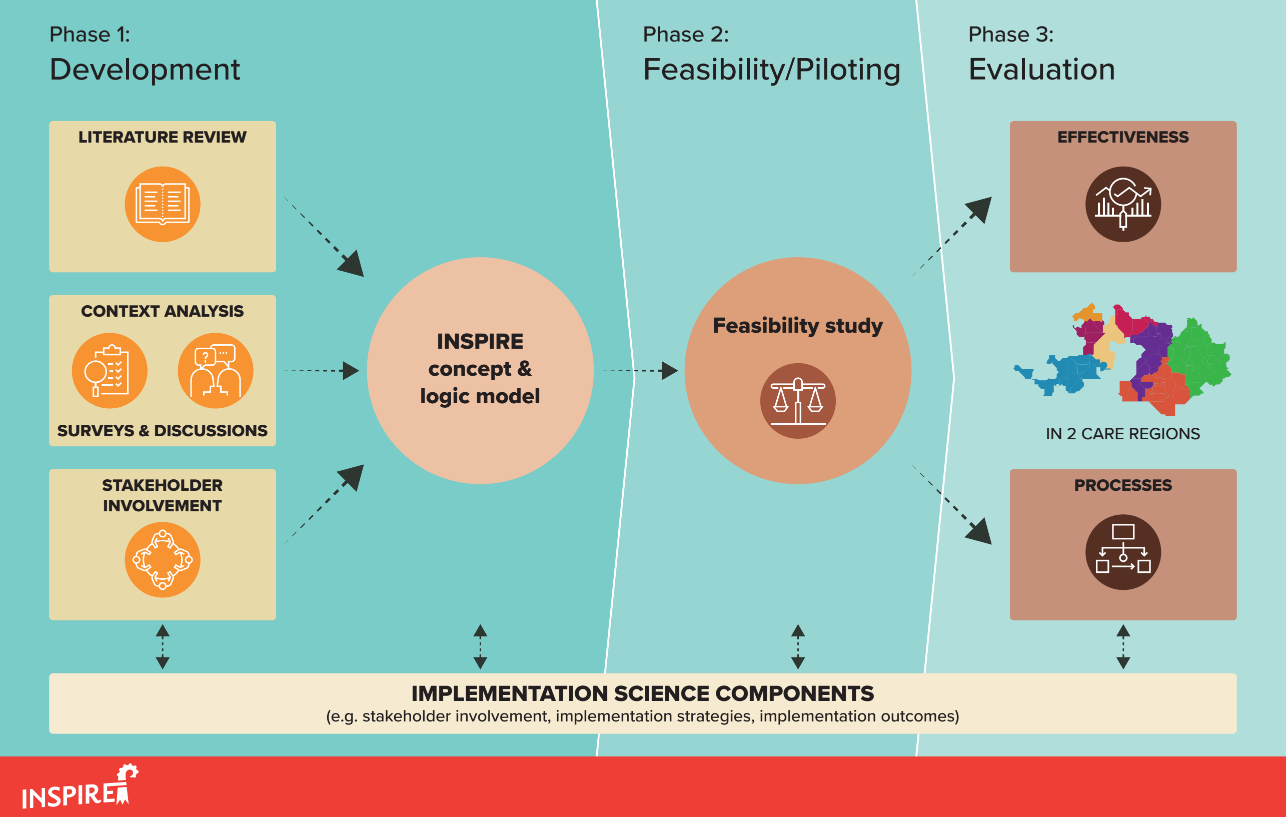

Phase 1:
Development
Phase 2:
Feasibility/Piloting
Phase 3:
Evaluation
LITERATURE REVIEW
CONTEXT ANALYSIS
SURVEYS & DISCUSSIONS
STAKEHOLDER INVOLVEMENT
INSPIRE concept & logic model
Feasibility study
EFFECTIVENESS
IN 2 CARE REGIONS
PROCESSES
IMPLEMENTATION SCIENCE COMPONENTS
(e.g. stakeholder involvement, implementation strategies, implementation outcomes)
INSPIRE
