# Supplementary material for: The INSPIRE Population Survey: development, dissemination and respondent characteristics
Source: BMC Med Res Methodol. 2021 Jun 24;21:131. doi: 10.1186/s12874-021-01329-3 (PMC8223353; doi:10.1186/s12874-021-01329-3)
Supplement: Supplementary file 2 — Additional file 2. The INSPIRE Population Survey in English. The file includes the INSPIRE Population Survey in English which had been provided for informative purposes and for which no backward translation was done [file 12874_2021_1329_MOESM2_ESM.pdf]

Who are you?

Not all older people want and need the same thing.

We would like to respond to your personal needs.

Therefore we would like to get to know you better!

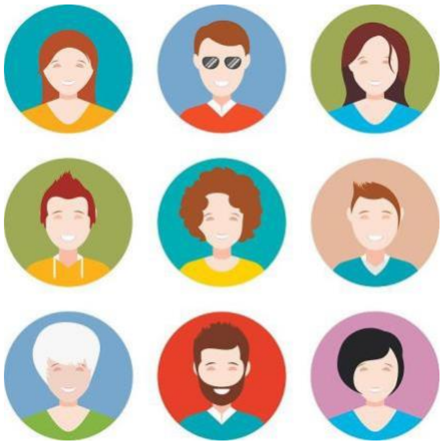

1. **Will you fill in the questionnaire alone or with the help of another person?**

   ☐ I'll fill it out alone.
   ☐ I'll fill it out with another person.

---

2. **Year of birth:** ☐☐☐☐☐

---

3. **Postcode of your place of residence:** ☐☐☐☐☐

---

4. **Sex:** ☐ Male Female ☐

---

5. **What country were you born in?**

   ☐ Switzerland
   ☐ France (France)
   ☐ Germany (German)
   ☐ Others: ____________________

---

6. **What is your highest level of education?**

- ☐ No school leaving certificate
- ☐ Elementary school
- ☐ Completed training
- ☐ Gymnasium
- ☐ University of Applied Sciences / Technical University
- ☐ University
- ☐ Don't know
- ☐ Others: ____

---

7. **How well do you speak German?**

- ☐ Swiss German is my mother tongue
- ☐ German is my mother tongue
- ☐ good
- ☐ Bad

---

8. **What is the current total monthly income of *your household* (all persons) in Swiss francs?**

☐ <3000 ☐ 3001-6000 ☐ 6001-9000

☐ >9000 ☐ Don't know ☐ I don't want to answer

---

9. **Do you have financial worries?**

☐ Yes ☐ No

---

10. **How are you insured with the health insurance?**

☐ General information ☐ Semi-private ☐Private☐ Flex

☐ No insurance

---

11. **Do you receive supplementary benefits?**

☐ Yes ☐ No. Don't know ☐

---

12. **Do you receive helplessness compensation?**

☐ Yes ☐ No Don't know ☐

How would you like to live?

For older people, housing is crucial for their quality of life.

Therefore, older people have more options to choose how they want to live.

We want to know what is important to you.

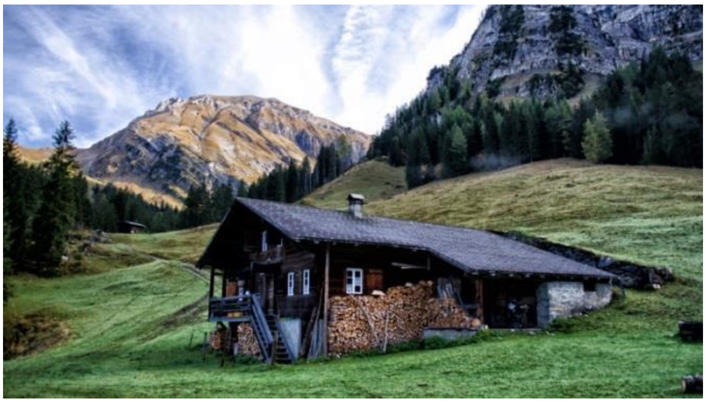

What is a good housing situation for you?

13. **What makes a good living situation for you?** *Please check all possible answers.*

- ☐ Your own apartment / house
- ☐ Barrier-free / wheelchair accessible
- ☐ Own garden
- ☐ Proximity to family and friends
- ☐ Proximity to nature / parks
- ☐ Proximity to the city
- ☐ Proximity to shopping facilities, library, etc.
- ☐ Proximity to doctors, health care facilities, etc.
- ☐ Proximity to church or social facilities
- ☐ Good public transport connections
- ☐ Opportunities for participation in social life
- ☐ Opportunities for exchange with other elderly people
- ☐ Opportunities for exchange with people of all ages
- ☐ Others:____

14. **What would be your ideal idea for living in old age *as long as you are independent*?** *Please tick only 1 answer.*

☐ In my own home *(please specify):*

  ☐ alone or with a partner
  ☐ with extended family
  ☐ with subtenant

☐ In the home of a family member (e.g. son, daughter)
☐ In a flat-sharing community
☐ In a flat for the elderly / assisted living
☐ In an old people's home / nursing home

---

15. **What would be your ideal idea for living in old age if you are *dependent on the support of other people* (e.g. Spitex, family, friends)?** *Please tick only 1 answer.*

☐ In my own home (*please specify*):

  ☐ alone or with a partner
  ☐ with extended family
  ☐ with subtenant

☐ In the home of a family member (e.g. son, daughter)
☐ In a flat-sharing community
☐ In a flat for the elderly / assisted living
☐ In an old people's home / nursing home

What is your current housing situation?

---

16. **My current housing situation is:** *Please tick only 1 answer.*

☐ Own house
☐ Rented house
☐ Own apartment
☐ Rented apartment
☐ old-age flat
☐ Other:__

---

17. **How many people live in your household (including you)?**

☐ ☐

18. **Who lives with you in the same household?** *Please check all possible answers.*

- ☐ I live alone
- ☐ (Spouse)Partner
- ☐ Other adults
- ☐ Siblings
- ☐ Adult children
- ☐ Professional help (e.g. a paid caregiver)
- ☐ Other:___________________

19. **Does your flat / house have anything of the following?** *Please check all possible answers.*

- ☐ Stairs **WITHOUT** handrail
- ☐ Stairs **WITH** handrail
- ☐ Stair lift
- ☐ Elevator
- ☐ Bath / WC on entrance level
- ☐ Bedroom or room used as a bedroom on the same floor.
- ☐ Wide door frames
- ☐ Trottoir in front of the house / apartment
- ☐ House / apartment is situated on a slope or on a hill

20. Are you generally satisfied with your current housing situation? *Please tick only 1 answer.*

- ☐ Yeah, I want to live as long as I can now.
- ☐ At the moment I am satisfied with my living situation, but I am planning to change it in the next few years
- ☐ No, I'd like to change my housing situation.

21. **What would you change?** *Please write.*

____

____

____

How much support do you need and receive in everyday life?

For most people, independence is important.

With increasing age, support is needed.

But support does not mean losing independence.

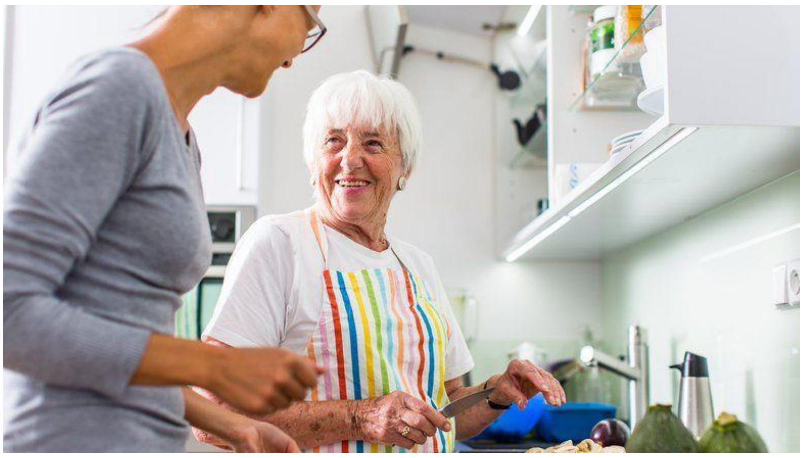

How independently can you move?

22. **Mobility:** *Please tick the box that best describes your health TODAY.*

- ☐ I don`t have **any** trouble walking around.
- ☐ I'm having a **little** trouble walking around.
- ☐ I have **moderate** problems walking around.
- ☐ I have **big** problems walking around.
- ☐ I'm **not gonna be able to** walk around

23. **Do you use the following aids?** *Please check all possible answers.*

☐ Walking stick	☐ Crutches	☐ Walker
☐ Rollator	☐ Wheelchair	☐ No aid
☐ Others: ______________________		

24. **Are you able to move completely independently outside the house? In** *the vicinity of the house or to neighbors.*

☐ Yes ☐ No

---

25. **What kind of transport do you currently use?** *Please check all possible answers.*

☐ I ride a bicycle / e-bike
☐ I drive my own car
☐ I use public transport
☐ I order and use a taxi on my own, but no public transport
☐ I use public transport in company
☐ I make limited journeys in a taxi or car in company
☐ I can't move outside the house anymore

---

26. **What kind of means of transport would you like to use in the future?** *Please check all possible answers.*

☐ bicycle
☐ e-bike
☐ My own car
☐ Bus or other public transport
☐ Help from friends or family
☐ taxi
☐ I don't intend to be mobile outside the house.
☐ Others: ______________________________

How independent are you when it comes to cooking and eating?

27. **Preparation of meals.** *Please tick only 1 answer.*

☐ I plan and cook meals **independently**
☐ I need **help preparing** meals
☐ I **warm up** the meals prepared by other people.
☐ The meals must be **prepared ready to eat.**

How independent are you when it comes to body care?

28. **Take care of yourself.** *Please tick only 1 answer.*

- ☐ I have **no** problems washing or dressing myself
- ☐ I have **slight** problems washing or dressing myself
- ☐ I have **moderate** problems washing or dressing myself
- ☐ I have **big** problems washing or dressing myself
- ☐ I'm **not able to** wash or dress myself.

---

29. **Are you able to go to the toilet on your own?**

☐ Yes ☐ No

How independent are you in managing your household?

30. **Everyday activities, e.g., housework, family or leisure activities**.
Please tick only 1 answer

- ☐ I don'**t** have **any** problems with my daily activities.
- ☐ I have **slight** problems with my daily activities.
- ☐ I have **moderate** problems with my daily activities.
- ☐ I have **big** problems to do my everyday activities
- ☐ I am not in a **position to** pursue my everyday activities

---

31. **Shopping.** *Please tick only 1 answer.*

- ☐ I can do **all my shopping independently**
- ☐ I can only do **small purchases independently**
- ☐ I need **help** shopping
- ☐ I'm **not able to do** any shopping.

---

32. **Do the laundry.** *Please tick only 1 answer.*

- ☐ I can wash the laundry **myself**
- ☐ I can do **small laundry**, e.g. socks, wash
- ☐ My laundry must be done **completely by others**

33. **Money budget.** *Please tick only 1 answer.*

- ☐ I manage financial transactions **independently** (budget, cheques, deposit, bank transfer)
- ☐ I can make the **daily, smaller expenses**, but I need help with transfers and bank transactions.
- ☐ I'm **no longer able to** handle money.

34. **Phone.** *Please tick only 1 answer.*

- ☐ I use the phone **independently**
- ☐ I'm just dialing some **known numbers**
- ☐ I pick up **the phone**, but I don't dial on my own.
- ☐ I don'**t** use the phone at all.

What support are you currently receiving?

35. **From whom do you receive regular support in everyday life?** *Please check all possible answers.*

- ☐ Family members of the same age (e.g. spouse, partner)
- ☐ Younger family members (e.g. children, grandchildren)
- ☐ Friends and neighbors
- ☐ I don't need

36. **From which organizations do you receive regular support in everyday life?** *Please check all possible answers.*

- ☐ Non-profit aid (e.g., Spitex)
- ☐ Private help (self-paid)
- ☐ Alzheimer's Association
- ☐ Parkinson's association
- ☐ Diabetes Association
- ☐ Red Cross Baselland
- ☐ Pro Senectute
- ☐ Other:_
- ☐ Other:_

37. **What kind of help did you need or did you use in 2018?**
Please check all possible answers.

- ☐ I didn't need any help in 2018.
- ☐ Care and support at your home *(please perform)*:
  - ☐ Public Organization
  - ☐ Private Organization
- ☐ Help with the housework *(please do)*:
  - ☐ Public Organization
  - ☐ Private Organization
- ☐ Meal service
- ☐ Physiotherapy
- ☐ Transport and assistance services (e.g. to the doctor, shopping)
- ☐ Day clinic
- ☐ Night clinic
- ☐ Old-age flat
- ☐ Short stays in old people's homes / nursing homes
- ☐ Others:______________________________

---

38. **How often is the following support from other people available to you?** *Whether by professional people or support from family / friends.*

a. Someone who will take you to the doctor if necessary.
☐ Never ☐ Sometimes ☐ Often ☐ Always

b. Someone who prepares food for you when you're not able to.
☐ Never ☐ Sometimes ☐ Often ☐ Always

c. Someone to help you with your day-to-day work when you're sick.
☐ Never ☐ Sometimes ☐ Often ☐ Always

d. Someone who can give you good advice in difficult situations.
☐ Never ☐ Sometimes ☐ Often ☐ Always

e. Someone you can trust or talk to about personal problems
☐ Never ☐ Sometimes ☐ Often ☐ Always

f. Someone who understands your problems.
☐ Never ☐ Sometimes ☐ Often ☐ Always

39. **Does the support you receive in everyday life meet your needs?**

☐ Yeah, I'm getting the support I need.

☐ No, I need more support.

40. **Do you look after, care for or support another person yourself? e.**g. children, elderly people, people with a disability

☐ Yes ☐ No

What about the support in the future?

41. **If you become more in need of help, which people do you prefer to receive regular support from in everyday life?** *Please check all possible answers.*

☐ Family members of the same age (e.g. spouse, partner)

☐ Younger family members (e.g. children, grandchildren)

☐ Friends and neighbors

42. **If you become more in need of help, which organizations do you prefer to receive regular support from in everyday life?** *Please check all possible answers.*

☐ Non-profit aid (e.g. Spitex)

☐ Private help (self-payment)

☐ Alzheimer's Association

☐ Parkinson's association

☐ Diabetes Association

☐ Red Cross Baselland

☐ Old Age and Nursing Home (APH)

☐ Pro Senectute

☐ Others:

☐ Others:

43. **If you become more needy, which of the following services would you consider?** *Please check all possible answers.*

☐ Care and support at your home *(please perform)*:

  ☐ Public Organization

☐ Private Organization

☐ Help with the housework (please do):

☐ Public Organization

☐ Private Organization

☐ meal service

☐ physiotherapy

☐ Transport and assistance services (e.g. to the doctor, for shopping)

☐ day clinic

☐ night clinic

☐ old-age flat

☐ Short stays in a retirement/nursing home

Don't know

Others: ____

44. **Would you consider the following technical aids or health technology in the future?**

a. **Telemedicine:** The possibility to communicate with your doctor via video or mobile phone.

☐ Yes

☐ Maybe

☐ No

☐ I don't understand what this technical aid is used for.

b. **Mobile phone or SMS service**: Applications / programs that remind you of your illness or provide information on how to manage your illness or take medication.

☐ Yes

☐ Maybe

☐ No

☐ I don't understand what this technical aid is used for.

c. **Portable device** (e.g. heart monitor, blood glucose monitor, SOS device, activity monitor)

☐ Yes

☐ Maybe

☐ No

☐ I don't understand what this technical aid is used for.

d. **Robot for help**, for use in the household and in care at home

☐ Yes

☐ Maybe

☐ No

☐ I don't understand what this technical aid is used for.

e. **Which of these technological tools or health technologies do you currently use?**

☐ telemedicine

☐ Mobile phone or SMS service

☐ Portable devices

☐ auxiliary robot

☐ I am not currently using any of these technical aids

How's your health?

Not all older people are equally healthy.

Health has a major impact on the needs of older people.

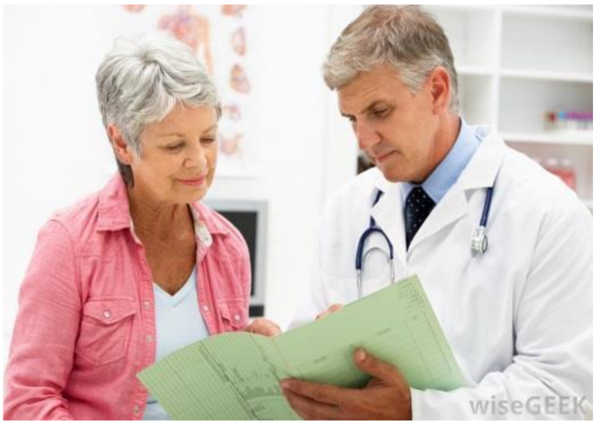

your senses

45. **Do you have problems in everyday life due to poor eyesight?**

☐ Yes ☐ No

46. **Do you have problems in everyday life due to bad hearing?**

☐ Yes ☐ No

nutrition

47. **Have you accidentally lost a lot of weight in the last 6 months?** (e.g. 3 kilograms in 3 months*)*

☐ Yes ☐ No

aches

48. **Do you regularly experience pain or discomfort?** *Please tick only 1 answer.*

☐ I have **no** pain or discomfort
☐ I have **slight** pain or discomfort
☐ I have **moderate** pain or discomfort
☐ I have **severe** pain or discomfort
☐ I have **very strong / unbearable** pain or discomfort

49. **Do you take painkillers regularly?** (e.g. Panadol / Dafalgan, Ibuprofen, Fentanyl, Tramal, Morphin)

☐ Yes, daily ☐ Yes, weekly ☐ No Do not know ☐

sleep

50. **How would you rate the overall quality of your sleep over the last four weeks?**

☐ Very good ☐Quite good ☐Quite poor ☐ Very poor Very poor ☐

51. **Do you regularly take sleeping pills?**

☐ Yes ☐No ☐ Don' t know

remembrance

52. **Do you have memory problems?**

☐ Yes No ☐

Do you regularly take medication?

53. **Are you currently taking four or more different medications?**

☐ Yes No ☐

54. **Do you need support to take your medication correctly?** *Please tick only 1 answer.*

☐ I am taking my medication on my **own in** exact dosage and at the correct time.

☐ I take **prepared** medications correctly (e.g. in dosettes)

☐ I cannot manage the correct intake of medication on my **own.**

What does your lifestyle look like?

How active are you?

How are you emotionally?

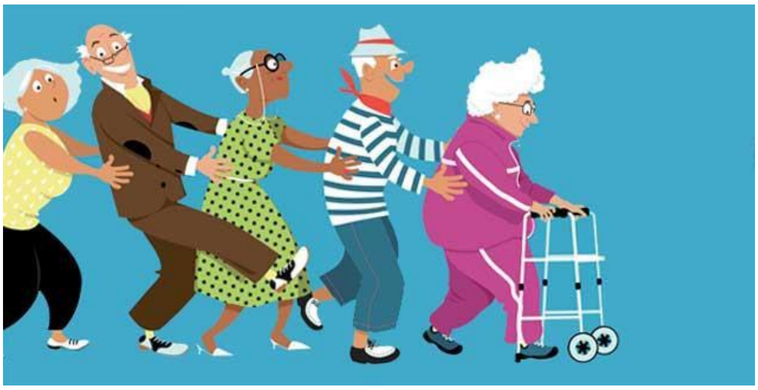

Lifestyle and well-being

55. **How many minutes of intense physical activity** *(e.g. jogging) do* **you exercise in a typical** ***week*****?**

☐ Less than 30 minutes
☐ 30-74 minutes
☐ 75 minutes or more

56. **How many minutes of lightly strenuous physical activity** *(e.g. walking S) do* **you exercise in a typical week?**

☐ Less than 30 minutes
☐ 30-74 minutes
☐ 75 minutes or more

57. **How often do you engage in physical activity that involves a combination of balance, muscle strengthening and endurance** *(e.g. dancing, yoga, tai chi, gardening)*?

☐ At least once a week
☐ Less than once a week
☐ Never

58. **If you had to rate your physical fitness with points from 0 to 10, where 0 means "very bad" and 10 "excellent", what score would you give yourself?** *Please circle a number.*

0 1 2 3 4 5 6 7 8 9 10
☹ ☺

59. **How often do you consume an alcoholic beverage?**

☐ Never
☐ 1 time a month or less
☐ 1 time per week or less
☐ 2 to 3 times a week
☐ 4 to 6 times a week
☐ Daily

60. **How many alcoholic beverages do you consume on a typical day you drink (a drink can be a glass of wine, a can of beer (355 ml) or 40 ml of spirits)?**

☐ 0 Beverages
☐ 1-2 drinks
☐ 3-4 drinks
☐ 5 or more drinks

61. **Do you currently smoke?**

☐ Yes, daily
☐ Yes, not every day
☐ No, but I was a smoker
☐ No, never

62. **Do you feel a general emptiness?**

☐ Yes ☐ Sometimes ☐ No

63. **Do you miss the company of other people?**

☐ Yes ☐ Sometimes ☐ No

64. **Do you feel abandoned?**

☐ Yes ☐ Sometimes ☐ No

65. **Have you been feeling miserable or depressed lately?**

☐ Yes ☐ No

66. **Have you been nervous or anxious lately?**

☐ Yes ☐ No

67. **How much do you feel anxious or depressed?** *Please tick only 1 answer.*

☐ I am **not** anxious or depressed
☐ I'm a **little** scared or depressed.
☐ I'm **moderately** anxious or depressed.
☐ I'm **very** anxious or depressed.
☐ I'm **extremely** anxious or depressed.

68. **Below you will find 3 statements. Please mark whether you agree or disagree with the individual statements.**

a. In many respects my life is almost ideal.

☐ Agreed ☐ Neither - nor ☐ I do not agree

b. My living conditions are excellent.

☐ Agreed ☐ Neither - nor ☐ I do not agree

c. I am satisfied with my life

☐ Agreed ☐ Neither - nor ☐ I do not agree

69. **In the first column, please specify the activities/groups for which you are active. Please indicate in the second column which activities / groups you are not participating in but would like to participate in.**

	Active in this	*Not active in it, but I want to be.*
Gymnastics clubs, sports groups	☐	☐
Hobby clubs, e.g. choir, music and theatre clubs	☐	☐
Professional associations, trade unions	☐	☐
Church congregation, religious groups	☐	☐
Political groups, parties	☐	☐
Self-help groups	☐	☐
Meeting with family, friends, neighbors	☐	☐
Voluntary work	☐	☐
Others: ___________________________	☐	☐

How often do you need medical attention?

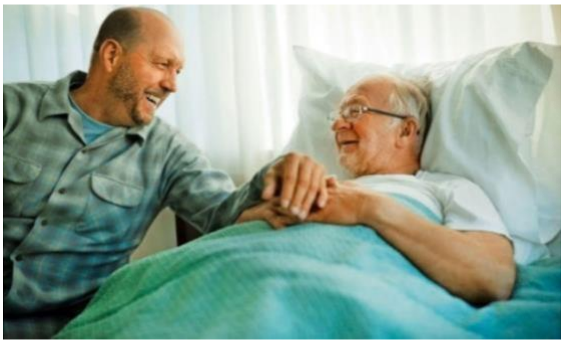

How often did you need medical help in 2018?

70. How many times did you visit your family doctor in 2018?

☐ ☐ times or ☐ more than 10

71. How often did you visit a specialist in 2018?

☐ ☐ times or ☐ more than 10

72. **How many times did you go to an emergency ward in a hospital in 2018?**

☐ ☐ times

73. **How many times were you in a hospital in 2018?** Just count the hospital stays you've had to spend the night in.

☐ ☐ times

74. **What other medical services did you use in 2018?**

______________________________ ______________________________
______________________________ ______________________________
______________________________ ______________________________

General health

75. **We want to find out how good or bad your health is TODAY. Please tick the box on the scale that best describes your health and enter the number you ticked on the scale in the box below.**

YOUR HEALTH TODAY =

☐☐☐☐

Best health,
that you can imagine

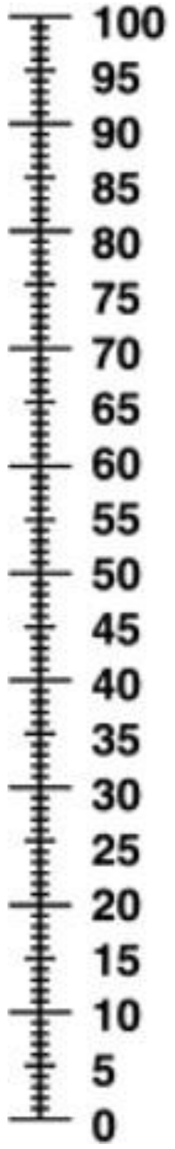

Worst health,
that you can imagine

Thank you very much for your time!
